# Bilaterally Symmetric Populations of Chicken dI1 (Commissural) Axons Cross the Floor Plate Independently of Each Other

**DOI:** 10.1371/journal.pone.0062977

**Published:** 2013-04-30

**Authors:** Keith D. Phan, Samantha J. Butler

**Affiliations:** Department of Biological Sciences, University of Southern California, Los Angeles, California, United States of America; King's College London, United Kingdom

## Abstract

Axons use temporal and directional guidance cues at intermediate targets to set the rate and direction of growth towards their synaptic targets. Our recent studies have shown that disrupting the temporal guidance process, by unilaterally accelerating the rate at which spinal dI1 (commissural) axons grow, resulted in turning errors both in the ventral spinal cord and after crossing the floor plate. Here we investigate a mechanistic explanation for these defects: the accelerated dI1 axons arrive in the ventral spinal cord before necessary fasciculation cues from incoming dI1 axons from the opposite side of the spinal cord. The identification of such an interaction would support a model of selective fasciculation whereby the pioneering dI1 axons serve as guides for the processes of the bilaterally symmetrical population of dI1 neurons. To test this model, we first developed the ability to “double” *in ovo* electroporate the embryonic chicken spinal cord to independently manipulate the rate of growth of the two bilateral populations of dI1 axons. Second, we examined the requirement for a putative bilateral interaction by unilaterally ablating the dI1 population in cultured explants of chicken embryonic spinal cord. Surprisingly, we find no evidence for a bilateral dI1 axon interaction, rather dI1 axons appear to project independently of each other.

## Introduction

During development, axons extend along stereotyped pathways to form the precisely ordered neuronal networks critical for the nervous system to function [1]. Axons are guided into and along these pathways by directional information present in the embryonic environment. These guidance signals orient axons by locally polymerizing or depolymerizing the actin cytoskeleton in the growth cone [2]. Our studies [3] have shown that there are also “temporal” guidance signals that regulate the speed of axon outgrowth by controlling the rate at which actin polymerizes, or “treadmills”, in the growth cone [4], thereby permitting neural circuits to develop in synchrony with the developing embryo.

The role for temporal guidance cues was first demonstrated for the MATH1^+^ (ATOH1) progenitor-derived dI1 neurons, a class of dorsal sensory interneurons in the developing spinal cord [5]. dI1 neurons differentiate adjacent to the roof plate (RP) at the dorsal midline and extend axons away from the RP, in response to a chemorepellent mediated by members of the Bone Morphogenetic Protein (BMP) family [6]. There are two populations of MATH1^+^ dI1 axons: 1) the well-described TAG1/axonin1^+^ commissural axons [7], [8], [9], [10] that project contralaterally, by crossing the ventral floor plate (FP) and turning rostrally towards the brain [11] and 2) a later born class of axons that project ipsilaterally, turning rostrally before reaching the FP [3], [12], [13]. Our studies have suggested that the BMPs, present in the RP, have multiple activities directing dI1 axon pathways: they both orient dI1 axons to extend away from the RP [6], [14] and locally limit the rate of dI1 axon extension through the dorsal spinal cord [3], [15].

Altering the rate at which dI1 axons grow had significant consequences for the development of the dI1 neural circuit. In particular, accelerating dI1 axon growth resulted in turning errors when dI1 axons reached the ventral spinal cord [3]. This observation suggested a previously unrealised level of control in the establishment of the dI1 neural circuit: that the speed of axon growth critically determines the response to guidance signals subsequently encountered along their route. However, the mechanistic basis for the “temporal” dI1 guidance errors has remained unresolved. One possibility was that the relevant directional guidance cues were not in place to guide accelerated dI1 axons through the ventral spinal cord, across the FP and towards the brain. Key molecular guidance signals for this segment of the dI1 trajectory include the diffusible cues, netrin1 [16], [17], SHH [18], [19], slit2 [20] and WNT4/WNT7b [21], and the contact mediated signals, F-Spondin [22], [23], NgCAM-related cell adhesion molecule (NrCAM) [24] and ephrinB4 [25], [26]. However, many of these cues, including SHH [27], netrin1 [16], [28], slit2 [20], NrCAM [29] and F-Spondin [23] have been shown to be present in chicken or rodent spinal cords prior to dI1 axons reaching the ventral spinal cord, suggesting that these guidance signals may, in fact, be in place to guide accelerated dI1 axons.

A second, largely unexplored, source of guidance cues in the ventral spinal cord could come from bilateral interactions between the two populations of dI1 axons projecting from either side of the spinal cord. Selective fasciculation between pioneer axons is common in invertebrates [30] but is not as well described in vertebrates. DI1 axons express several cell adhesion molecules (CAMs), including TAG1/axonin1 [24], [31], and Neuron-glia cell adhesion molecule (NgCAM) [32], which could provide a substrate to encourage axon growth. The turning errors were observed when only one of the bilaterally symmetric populations of dI1 axons was accelerated by *in ovo* electroporation of chicken embryos. In these embryos, 1) accelerated early born contralaterally-projecting dI1 axons would have no bilateral axon interactions in the FP and 2) accelerated later born ipsilaterally-projecting dI1 axons would not encounter the opposing population of earlier born contralaterally-projecting dI1 axons as they navigated the ventral spinal cord. We have thus explored whether these two putative bilateral axonal interactions have a critical role in the guidance of dI1 axons, using chicken embryos as a model system. We first developed a “double” electroporation technique to examine whether the previously observed defects after unilateral acceleration of one population of dI1 axons can be rescued by the bilateral acceleration of both populations of dI1 axons. Second, we used an *in vitro* tissue culture assay to assess the effect of unilaterally ablating dI1 neurons on the axon trajectory of the spared population of dI1 axons on the other side of the spinal cord. Neither experiment provided any evidence for a bilateral interaction between dI1 axons, suggesting that the two populations of axons project independently of each other.

## Materials and Methods

### Double *in ovo* Electroporation

Hamburger-Hamilton (HH) stages 14/15 White Leghorn chicken embryos (McIntyre Poultry) were injected with two sequential solutions of different combinations of the following expression constructs: 0.25 µg/µl Math1::tandem dimer (td) *tomato,* 0.25 mg/ml Math1::farnesylated (f) *Gfp* and 0.6 µg/µl Math1::*BmprII*Δ*Lim-Gfp*. When two fluorophores are electroporated into dI1 neurons using the Math1 enhancer, there is close to a 100% overlap between the expression of the fluorophores. In no case, did double electroporation alter the number of LHX2/9 dI1 neurons.

#### First injection

DNA solution #1 was injected into the lumen of the spinal cord and an electric current passed across the embryo using a BTX Electro Square Porator (ECM 830) set at ten 50 ms second pulses of 30 V. The embryo was permitted to settle for 10–15 minutes and the positive and negative electrodes were inverted.

#### Second injection

DNA solution #2 was electroporated into the lumen of the spinal cord using five 50 ms pulses of 30 V. A few drops of 1× penicillin/streptomycin/glutamine (PSG, Invitrogen) were applied to the embryo, and the egg was wrapped in parafilm (VWR) and incubated at 37°C for 2 days until HH stages 24/25.

### Ablated “Open Book” Tissue Culture Assays

HH stages 14/15 chicken embryos were electroporated with 0.25 mg/ml Math1::f*Gfp* or 0.6 mg/ml Math1::*BmprII*Δ*Lim-Gfp* into the developing spinal cord lumen using an electric current generated by five 50 ms pulses of 30 V. The electroporated egg was incubated overnight at 37°C and then dissected in L15 medium (CellGro) at HH stage 18, before any dI1 commissural axons have reached the FP [3]. Using a Zeiss M2 Bio fluorescence dissection microscope to visualize the electroporated side, the dorsal half of the non-electroporated side of the embryonic spinal cord was removed. The remaining “open book” explant of spinal cord was embedded in a collagen matrix [33] and incubated in Neurobasal medium (Invitrogen) +1xPSG at 37°C for another 30 hours i.e. to approximately HH stage 24/25.

### Immunohistochemistry and Quantification of Open Book Explants

All spinal cords were processed to result in fixed “open book” explants embedded in collagen. Antibody staining was as previously described [6]. Fluorescence images were collected on a Carl Zeiss LSM510 confocal and Axioplan 2 microscope. Images were processed using Adobe Photoshop CS4.

Antibodies against the following proteins were used. Rabbit: LHX2/9 (pan LH2A/B), 1∶1000 [34]; islet1/2 (K5), 1∶2000 [35]; axonin1 [36], 1∶10,000; Mouse: GFP, 1∶1000 (3E6, Invitrogen). Species appropriate Cyanine 3 and Fluorescein conjugated secondary antibodies were used (Jackson ImmunoResearch Laboratories).

These explants were quantified by comparing the number of ipsilaterally projecting GFP^+^ axons, i.e. those that normally turn rostrally before reaching the FP, to the number of contralaterally projecting GFP^+^ axons, i.e. those that normally turn rostrally after crossing the FP. Fluorescent images were collected on Carl Zeiss LSM510 confocal and Axiovert 200 M microscopes. Images were processed using Adobe Photoshop CS4.

## Results

### Double *in ovo* Electroporation can Differentially Label the Bilaterally Symmetric Populations of dI1 Neurons

**Figure 1:**
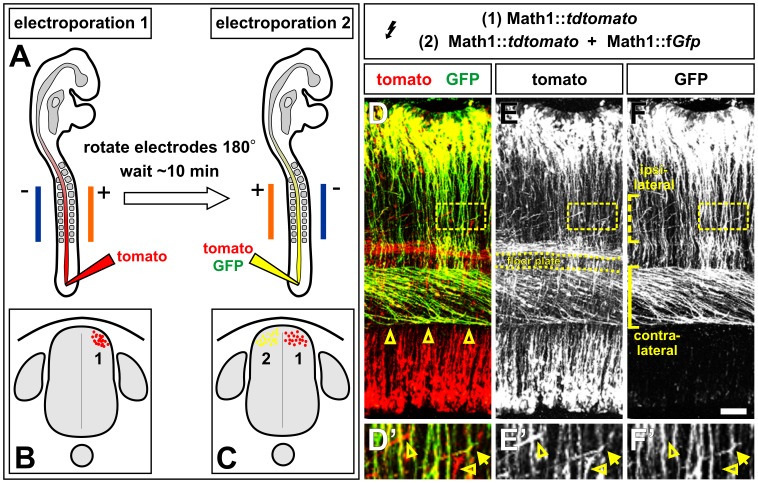
Double in ovo electroporation permits the independent manipulation of bilateral commissural axon trajectories. (A–C) Schematic diagram illustrating the double in ovo electroporation procedure. In the first electroporation, a single fluorophore, here tandem dimer (td) tomato [51], is introduced into HH stages 14/15 chicken spinal cords using a double dose of electrical pulses (B). The electrodes are then rotated 180°. After a brief pause, two fluorophores, tdtomato and farnesylated (f) GFP, are introduced into the spinal cord using a single dose of pulses in a second electroporation. If the fluorophores are delivered under the control of the Math1 enhancer [37], this strategy will result in the bilateral symmetric populations of commissural neurons being differentially labeled either red or yellow (C). (D–E) Longitudinal “open book” preparations from HH stage 23/24 double in ovo electroporated chicken spinal cords. The trajectory of the tomato+ GFP+ (yellow) commissural axons can be easily distinguished against the ubiquitous background of tomato+ (red) commissural axons. There are two classes of yellow (and red) ventrally projecting commissural axons: an ipsilateral population that turns before the floor plate (FP, dotted bracket, F) and a contralateral population that makes a sharp rostral turn after crossing the FP (open arrowheads, D; solid bracket, F). (D’–F’) Higher magnification images of the boxed region in D–F show that the yellow ipsilateral population of commissural axons (arrow) can easily be distinguished from the red contralateral population (open arrowheads’). Scale bar: 60 µm.

As a first step towards assessing the role of putative interactions between the axons of the bilaterally symmetric populations of spinal MATH1^+^ dI1 neurons, we developed a novel method of independently labeling axons on different sides of the spinal cord by “double” *in ovo* electroporating chicken embryos. In control experiments to establish this procedure, HH stages 14/15 chicken embryos were electroporated twice, first with Math1::td*tomato* and then with a mixture of both Math1::td*tomato* and Math1::f*Gfp*, reversing the position of electrodes between the injections (Fig. 1A). One fluorophore was always electroporated into both sides of the spinal cord, to avoid having to remove the remnants of DNA between electroporations. To ensure the success of the first electroporation, the dose of electric current delivered in the first electroporation was double that of the second electroporation (Figs. 1B, C). The MATH1 enhancer directs the expression of genes to dI1 progenitors [37]. Labeled dI1 axons project either ipsilaterally, turning rostrally before reaching the FP, or contralaterally, turning rostrally after crossing the FP (Fig. 1F) [3], [13].

The double electroporation procedure results in tomato being introduced into all dI1 axons (Fig. 1D, E), while GFP is present in dI1 axons on only one side of the spinal cord (Figs. 1D, F). This distribution of fluorophores permits us to easily distinguish where a dI1 axon originated from within the spinal cord. This distinction is particularly critical in the ventral spinal cord, where a mixture of ipsilateral and contralateral populations of dI1 axons turn rostrally. After double electroporation, one side of the ventral spinal cord will contain a tomato^+^/GFP^+^ (i.e. yellow) ipsilateral dI1 axon population (arrow, Figs. 1D’–F’) and a tomato^+^ contralateral dI1 axon population (open arrowheads Figs. 1D’–F’). The situation is reversed on the other side of the spinal cord.

### Bilateral Acceleration of dI1 Axons does not Rescue the Unilateral Acceleration Phenotype

We next compared the consequence of either unilaterally or bilaterally accelerating dI1 axons. Our previous experiments have shown that introducing a truncated form of the type II BMP receptor (BMPRIIΔLim-GFP) into dI1 neurons results in their extending axons that grow 40% faster than axons expressing GFP alone [3]. This accelerated rate of growth had significant consequences for the fidelity of subsequent guidance decisions: there was an 80% decrease in the number of ipsilaterally projecting dI1 axons and 4-fold increase in the number of contralaterally projecting dI1 axons that aberrantly turn caudally away from the brain [3].

**Figure 2:**
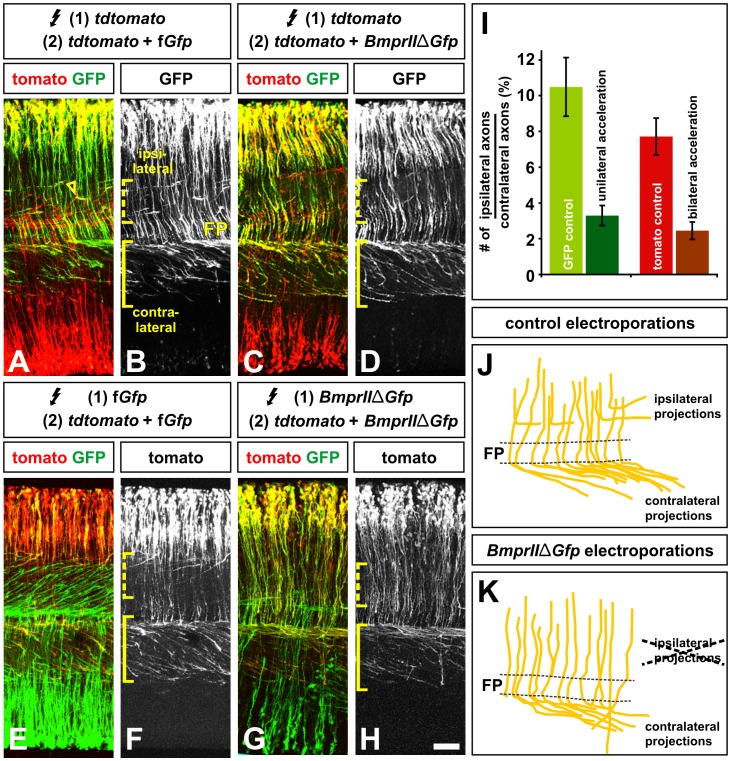
Accelerating axon growth either unilaterally or bilaterally results in the same turning errors. (A–I) To compare the consequence of either unilaterally or bilaterally accelerating commissural axon growth, chicken embryos were electroporated at HH stages 14/15 and longitudinal open book preparations of the spinal cord generated at HH stages 23/24. All expression vectors were electroporated under the control of the Math1 enhancer. Only the behavior of the yellow GFP^+^ tomato^+^ commissural axons was monitored (A–H) and quantified (I). (A, B, E, F, J) By HH stages 23/24, control neurons electroporated with both fGFP and tomato (yellow), in either a control tomato background (A, B) or a control GFP background (E, F) project axons ventrally and then sharply rostrally, to extend towards the brain. There are two classes of yellow axons, an ipsilateral population that turns before the FP (dotted bracket, B, F) and a contralateral commissural population that turns after crossing the FP (solid bracket, B, F). Note that the ipsilateral axons often turn on apparently encountering a contralateral dI1 axon (arrow, A). (C, D, K) In contrast, by the same stage, commissural neurons unilaterally electroporated with BMPRIIΔLim-GFP in a control tomato^+^ background, project very few axons ipsilaterally (dotted bracket, C) and the contralaterally projecting commissural axons turn both rostrally and caudally (solid bracket, C). (G, H, K) Commissural neurons electroporated with tomato in a bilaterally accelerated BMPRIIΔLim-GFP^+^ background make very similar turning errors to those observed after unilateral acceleration (C, D). (I) Control commissural axons (either GFP^+^ in a tomato^+^ background or tomato^+^ in a GFP^+^ background) project ipsilaterally to a statistically similar extent (p>0.078, Student’s *t*-test; GFP^+^ axons: 10.5%±1.3 turn ipsilaterally, n = 3698 total axons in 18 open book preparations; tomato^+^ axons: 7.7%±1.0, n = 1557 axons in 11 open book preparations). In contrast, far fewer axons turned ipsilaterally after either unilateral or bilateral acceleration of commissural axons (unilateral acceleration: 3.3%±0.55, n = 1321 axons in 6 open book preparations, p<0.003 statistically similar to control GFP^+^ axons; bilateral acceleration: 2.4%±0.49 n = 1108 axons from 6 open book preparations, p<0.01 statistically similar to control tomato^+^ axons). There is no statistical difference between the phenotype observed after unilateral or bilateral acceleration of commissural axons (p>0.13). Scale bar: 60 µm.

We first repeated this experiment in the context of the double *in ovo* electroporation procedure. To unilaterally accelerate dI1 axons, BMPRIIΔLim-GFP was introduced into only one population of dI1s, whereas tomato was introduced bilaterally in all dI1s (Fig. 2C, D). The effect of BMPRIIΔLim-GFP on dI1 axon extension was compared to control experiments where GFP was unilaterally introduced into dI1 neurons, in a tomato^+^ background (Fig, 2A, B). This experiment gave similar results to those observed previously [3]: there was an ∼70% decrease in the number of accelerated BMPRIIΔLim-GFP^+^ tomato^+^ ipsilaterally projecting dI1 axons (dotted bracket, Fig. 2D, I, K) compared to the number in GFP^+^ tomato^+^ controls (dotted bracket, Fig. 2B, I, J).

If these turning errors result from accelerated dI1 axons reaching the ventral spinal cord before the bilateral interactions between dI1 axons is possible, we reasoned that we might observe a reduction in guidance errors when both populations of dI1 axons are concomitantly accelerated. To examine this hypothesis, tomato was introduced unilaterally into dI1 axons, in a background of either GFP (control, Fig. 2E, F) or BMPRIIΔLim-GFP (bilateral acceleration, Fig. 2G, H). However, no significant rescue of the turning phenotypes was observed, rather there was a 70% decrease in the number of accelerated tomato^+^ BMPRIIΔLim-GFP^+^ ipsilaterally projecting dI1 axons (dotted bracket, Fig. 2H, I, K) compared to the number in tomato^+^ GFP^+^ controls (dotted bracket, Fig. 2F, I, J). A significant subset of the contralateral dI1 axons was observed to project caudally (bracket, Fig. 2H), suggesting that this phenotype was also not rescued.

### Unilaterally Ablating Commissural Neurons does not Affect the Trajectories of the Contralateral Side

**Figure 3:**
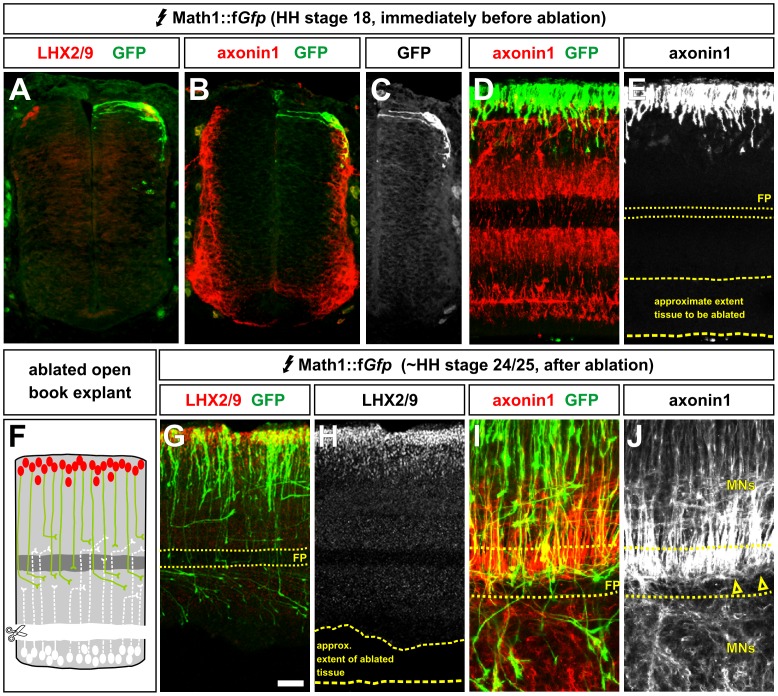
dI1 neurons can be unilaterally ablated in open book preparations of the spinal cord. (A–E) Chicken embryos were electroporated with a Math1::f*Gfp* expression vector at HH stages 14/15 and incubated until HH stage 18. To assess the extent of axon outgrowth transverse cross sections (A–C) or longitudinal open-book preparations (D, E) were labeled with antibodies against either LHX2/9 (A), which specifically labels dI1 nuclei [52], [53] or axonin1 (B–E), which labels pre-crossing commissural axons as well as some motor axons [15]. At this stage, none of the dI1 GFP^+^ axons (E) and very few of the axonin1^+^ commissural axons (D) have extended beyond the dorsal region that will be ablated. The axonin1^+^ processes in the ventral spinal cord appear to be emanating largely from the motor column. (F) The dorsal half of the spinal cord was unilaterally ablated at HH stage 18. Open book explants of the spinal cord were then cultured until ∼HH stages 24/25. (G–J) To assess whether the ablation of dI1 neurons was successful, the open-book preparations were labeled with antibodies against LHX2/9 (G, H) or axonin1 (I, J), which labels pre-crossing commissural axons as well as some motor axons [15]. In both cases, there was no evidence of any LHX2/9^+^ axonin1^+^ dI1 neurons remaining on the ablated side (H, J). Note that there is residual axonin1 staining in the motor neurons (MN) on both the unoperated and operated sides. Scale bar: (A–C) 30 µm; (D, E) 55 µm; (G, H) 70 µm; (I, J) 25 µm.

**Figure 4:**
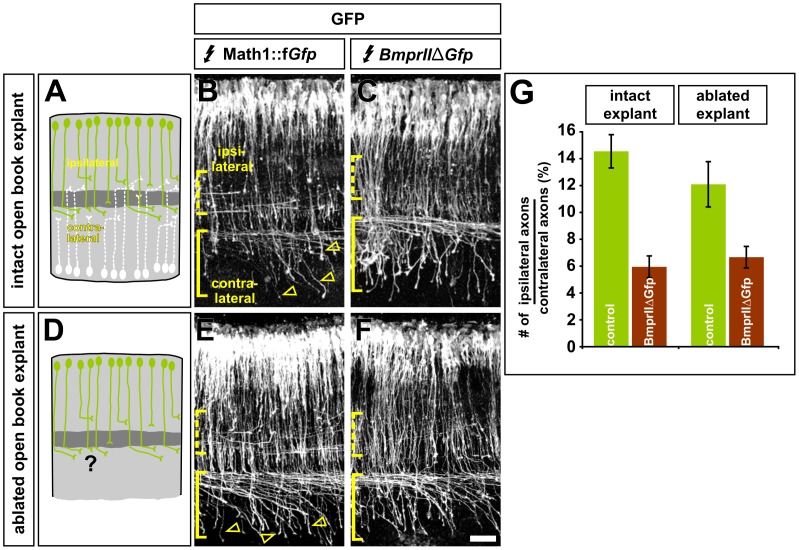
The unilateral ablation of dI1 neurons does not affect the turning behaviors of the remaining contralateral population of dI1 axons. (A–F) To determine the consequence of unilaterally ablating dI1 neurons on the trajectory of the contralateral population of dI1 neurons, chicken embryos were electroporated at HH stages 14/15, incubated until HH stage 18 and then cultured as either intact (A) or unilaterally dorsally-ablated (D) open book explants of the spinal cord until ∼HH stages 24/25. All expression vectors were electroporated under the control of the Math1 enhancer. (B) Control GFP^+^ dI1 axons projected relatively normally in the cultured explant, with axons projecting both ipsilaterally (dotted bracket) and contralaterally (solid bracket). (C) As observed in intact embryos, accelerated BMPRIIΔLim-GFP^+^ axons project very few axons ipsilaterally (dotted bracket) compared to those projecting contralaterally (solid bracket) (D) Ablating the opposing population of dI1 neurons has no apparent effect on the projection patterns of control GFP^+^ axons. (E) Similarly, the ablation had no obvious effect on the trajectories of accelerated BmprIIΔLim-GFP^+^ axons. (F) Control GFP^+^ dI1 axons project ipsilaterally to a statistically similar extent (p>0.12, Student’s *t*-test) in both intact (21.4%±2.3, n = 1563 total axons in 12 explants) or ablated (15.6%±2.5, n = 2032 axons in 11 explants) open book explants. In contrast, fewer axons turned ipsilaterally in either the intact (7.1%±0.8, n = 2120 total axons in 8 explants) or ablated (8.2%±1.1, n = 3058 axons in 12 explants). There is no statistical difference between the phenotype observed after dI1 axon acceleration in intact or ablated explants (p>0.27). Scale bar: 60 µm.

Since the restoration of any bilateral dI1 axon interactions was not sufficient to rescue the defects in turning behavior observed after accelerating dI1 growth, we next examined whether the putative bilateral interaction was required for normal turning behaviour. To examine this question, we developed an *in vitro* culture assay using explants of chicken spinal cord. Chicken embryos were electroporated with either Math1::f*Gfp* or Math1::BmprIIΔLim-GFP at HH stages 14/15 and incubated *in ovo* until HH stage 18. At these stages, MATH1^+^ dI1 axons have not extended past the intermediate spinal cord (Fig. 3A, C–E) [3]. The embryos were then dissected to produce either control intact open book explants of the spinal cord (Fig. 4A) or explants in which the dorsal spinal cord on one side of the explant had been removed (Fig. 4D). These explants were then cultured for another 30 hours until they reached approximately HH stage 24. The ablation of dI1 neurons was successful, since no LHX2/9^+^ dI1 nuclei or dorsal axonin1^+^ commissural axons were observed on the ablated side of the open book preparations (Fig. 3G–J). Axonin1^+^ commissural axons grew across the FP only from the unoperated side of the open book explant (arrowheads, Fig. 3J) and axonin1 expression was extinguished on the postcrossing commissural axons, as has been reported for its mammalian homolog, TAG1 [38].

The control GFP^+^ dI1 axons project with remarkably similar trajectories in both the intact and the ablated explants. 12–14% of dI1 axons turn ipsilaterally compared to those projecting contralaterally, a figure that is statistically similar to dI1 axons extending *in vivo* (Fig. 2I [3]). On the contralateral side, although many dI1 axons make the sharp rostral turn normally observed *in vivo*, about 25% of GFP^+^ axons that extend across the FP do not turn at all, rather they continue growing straight (arrowheads, Fig. 4B, E). However, this phenotype is seen with similar frequency in both control (Fig. 4B) and ablated (Fig. 4E) GFP^+^ preps, suggesting that it is a result of the experimental procedure, possibly revealing a requirement for a repulsive boundary in the ablated tissue, rather than a consequence of the ablation. Supporting the idea that there is no interaction between the opposing populations of dI1 axons, we found that there was no significant difference between the phenotype of accelerated axons in intact (Fig. 4C) verses ablated (Fig. 4F) explants, i.e. there was no further decrease in the number of ipsilaterally turning axons (Fig. 4G).

Taken together, this data shows that the presence of dI1 axons is neither sufficient nor required for the turning behavior of the dI1 axons on the opposing side of the spinal cord, strongly suggesting that there is no guidance interaction between them.

## Discussion

### The Opposing Populations of dI1 Axons do not Appear to Interact in the Ventral Spinal Cord

Many studies have demonstrated that selective fasciculation between axons has a critical role during the formation of neural circuits, particularly in invertebrates [30]. A key example of this mechanism is that pioneering axons from early born neurons are thought to act as a scaffold for the axons from later born neurons [39], [40]. Selective fasciculation is often mediated through contact dependent pathways: CAMs on the surface of the axons dictate the homophilic or homophobic interactions that direct the axons towards or away from potential fasciculation partners. Although it seems likely that selective fasciculation between axons is an evolutionarily conserved guidance mechanism, its role in directing the path of spinal commissural axons has remained unclear [41].

Studies over many years have shown that the contralateral dI1 (commissural) axon pathway in the spinal cord is guided in part by heterophilic interactions between CAMs in the axons themselves (axonin1/TAG1, NgCAM) and in the cells of the FP (NrCAM, F-Spondin). TAG1 has a neurite promoting activity *in vitro* [31]. Blocking axonin1 or NrCAM results in commissural axons failing to cross the FP, suggesting that positive heterophilic interactions between these CAMs are required for passage across the FP [24], [32]. Perturbing NgCAM resulted in the defasciculation of commissural axons but did not otherwise alter their trajectory across the FP, making its role less clear [32].

These studies focused on the contact-mediated interactions between the commissural axons and the cells of the FP and did not examine the role of putative interactions between the opposing populations of commissural axons. Thus, it remained unresolved whether the *in vivo* phenotypes observed after blocking axonin1 or NrCAM are only a consequence of interactions between heterophilic CAMs or whether there was also a homophilic component resulting from a bilateral interaction between the different populations of dI1 axons growing within the FP and the ventral spinal cord. The close proximity of dI1 commissural axons extending across the FP and the co-incidence of the ipsilateral and contralateral dI1 axons growing within the ventral spinal cord make such an interaction feasible [11]. For example, the pioneer TAG1/axonin1^+^ commissural axons entering the FP could provide a positive substrate for one another, while the rostral turn taken by the contralateral dI1 axons could provide a scaffold for the rostral turn of the later born ipsilateral dI1 axons (arrow, Fig. 2A).

Nonetheless, we were unable to find any evidence for such bilateral interactions between the opposing populations of dI1 axons. Restoring the bilateral interaction was not sufficient to rescue the defects seen after unilaterally accelerating the rate of dI1 axon growth. Moreover, ablating one population of dI1 neurons did not affect the turning behaviors of the remaining dI1 axons. Taken together, these data suggest that the turning behaviors of dI1 axons in the FP and ventral spinal cord occur independently from one another.

### Potential Mechanisms for Temporal Guidance Errors

Our studies [3], [42], and those of others [43], [44], [45], [46], [47], [48] have shown that guidance factors can regulate the rate of axon extension by controlling the activity of cofilin and its negative regulator Limk1. Cofilin acts to depolymerize or sever actin [4]. These severed actin monomers are the preferred substrates for further rounds of actin polymerization, thus dynamic treadmilling of actin only occurs when there is a balance between the activation states of Limk1 and cofilin. Limk1 activity is regulated by the BMP signaling pathway [44], [49]. In our studies, the truncated version of BMPRII, BMPRIIΔLim-GFP, appears to act solely through the cofilin/Limk1 pathway: introducing BMPRIIΔLim-GFP in dI1 neurons lowered the activity of Limk1 and accelerated axon outgrowth, such that dI1 axons grew up to 40% more rapidly and made guidance errors in the ventral spinal cord [3]. These effects were phenocopied by directly elevating the level of cofilin in dI1 neurons [3].

The mechanistic basis for these guidance errors remains unresolved. Temporal guidance cues could permit axons to encounter directional information at the right time in development, such that neural circuits develop in concert with the rest of the embryo. However, as discussed above, many of the key molecular guidance cues are present in the FP prior to dI1 commissural axons reaching the ventral spinal cord. A notable exception is WNT4, which is expressed in the ventral spinal cord by HH stages 27/28 [50] and controls the contralateral rostral turn towards the brain [21]. Future studies will determine whether the effect of ectopic early WNT expression on the growth of accelerated dI1 axons. A second possibility is that axons must navigate intermediate targets at particular speeds, similar to a car altering speed as it negotiates a curve in the road. If axon growth is constitutively accelerated, the fidelity of a guidance decision may be lessened with some axons “derailing” at the choice point. Further examination of the behavior of first, accelerated dI1 growth cones as they navigate intermediate targets and second, other classes of accelerated axons, will be instructive in resolving these issues.

### Conclusions

In summary, these studies show a surprising absence of any interaction between the two opposing populations of dI1 axons in the spinal cord as they navigate the ventral spinal cord.
